# Varicella zoster virus-associated Chorioretinitis: a case report

**DOI:** 10.1186/s12886-018-0696-3

**Published:** 2018-02-05

**Authors:** Joo Yeon Kim, Ji Hwan Lee, Christopher Seungkyu Lee, Sung Chul Lee

**Affiliations:** 1 Department of Ophthalmology, The Institute of Vision Research, Yonsei University College of Medicine, Yonsei-ro 50-1, Seodaemun-gu, Seoul, South Korea; 2 Department of Ophthalmology, National Health Insurance Service Ilsan Hospital, Goyang, South Korea

**Keywords:** Chorioretinitis, Herpesvirus 3, Human, Uveitis

## Abstract

**Background:**

Chorioretinitis is an unusual form of varicella zoster virus (VZV)-associated uveitis, and no report has described VZV-associated chorioretinitis using serial optical coherence tomography (OCT) images obtained during the course of resolution.

**Case presentation:**

A 61-year-old woman presented with acute, unilateral vision loss in her right eye. Her visual acuity was count fingers in the right eye and 16/20 in the left eye, and she exhibited skin vesicles on her right forehead. Slit lamp biomicroscopy, funduscopy, OCT, and intraocular fluid analysis were performed. The right eye exhibited multiple inflammatory lesions at the posterior pole, macular edema, and disc swelling on the fundus examination. OCT revealed predominant involvement of the choroid and the retinal pigment epithelium (RPE). Intraocular fluid analysis showed positivity for VZV. The patient was admitted and treated with intravenous acyclovir. Additional oral prednisolone was used to reduce the inflammatory reaction. After 2 weeks of treatment with acyclovir, the lesion resolved, with undulation of the RPE. Her final visual acuity was 20/20.

**Conclusions:**

VZV-associated posterior uveitis may present as multifocal chorioretinitis. Intraocular fluid analysis is important to detect an infectious origin.

## Background

Varicella zoster virus (VZV) may involve the posterior segment of the eye and is an important cause of acute retinal necrosis (ARN), which is characterized by rapidly progressing peripheral necrotizing retinitis and a prominent inflammatory reaction in the vitreous/anterior chamber [1]. VZV has been also associated with the non-ARN type of posterior uveitis or multifocal posterior necrotizing variants [2, 3]. Chorioretinitis is an unusual form of VZV-associated uveitis [3–6]. Herein, we describe a case of VZV-associated chorioretinitis using multimodal images.

## Case presentation

**Fig. 1:**
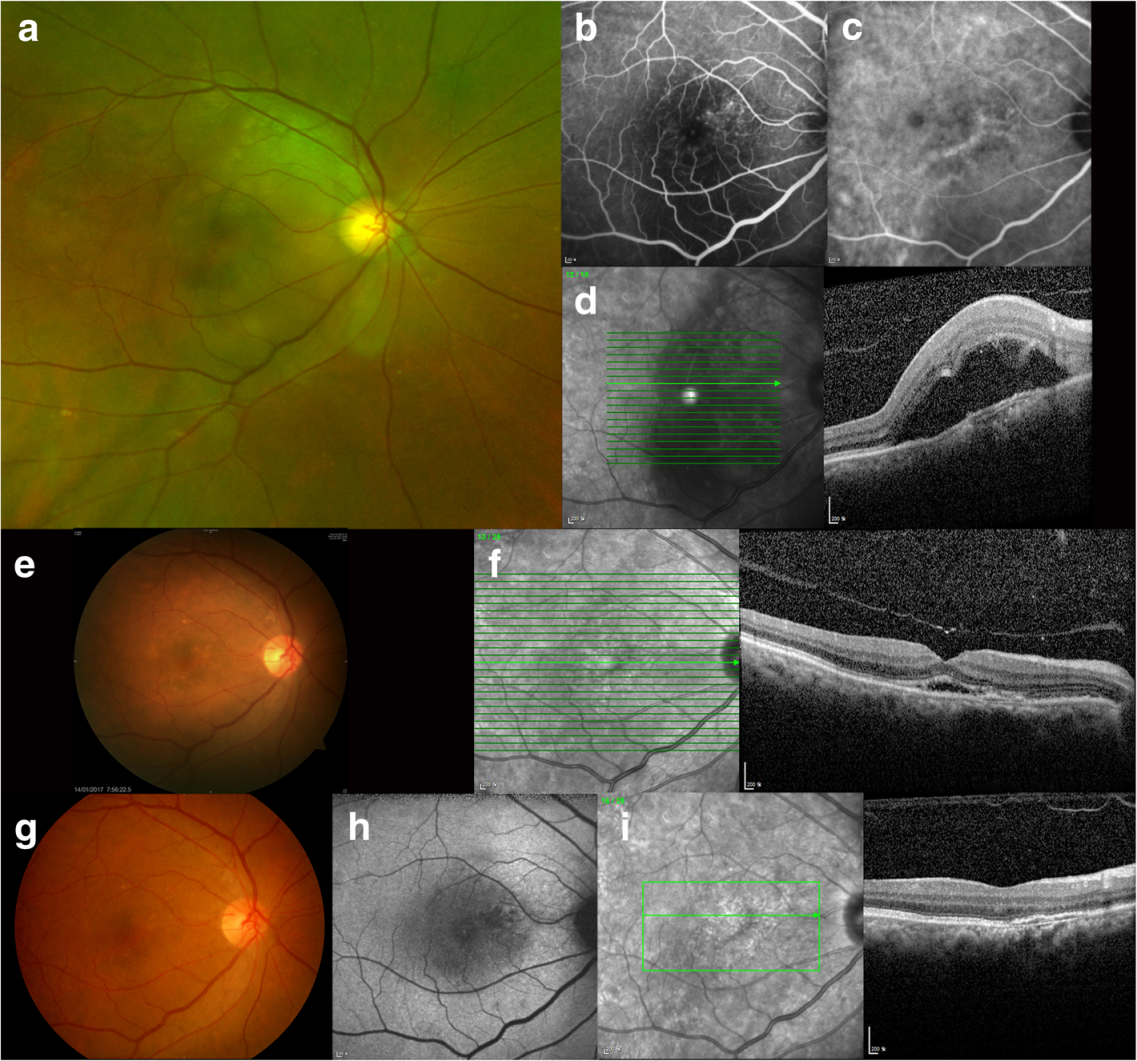
Imaging findings for a 61-year-old woman with varicella zoster virus-associated chorioretinitis. Multiple chorioretinal lesions can be observed superonasal to the fovea at the initial visit (**a**). Fluorescein and indocyanine green angiography findings in the late phase (**b**, **c**). Optical coherence tomography reveals hyper-reflective spots in the choroid layer and focal thickening of the choroid with an irregular overlying retinal pigment epithelium (**d**). Fundus photography and optical coherence tomographic images at 1 week after intravenous acyclovir treatment (**e**, **f**). The chorioretinal lesion and macular edema have resolved, although focal hypo-reflectivities observed on fundus autofluorescence remain because of atrophic changes at 2 weeks after intravenous acyclovir treatment (**g**–**i**)

A 61-year-old woman presented with acute vision loss in her right since 1 day. Her medical history included hypertension, cerebral aneurysm, and Alzheimer’s dementia. She was on oral acyclovir for herpes zoster infection involving the trigeminal nerve for a week. At the initial visit, her visual acuity was count fingers in the right eye and 16/20 in the left eye. The corresponding intraocular pressure values were 13 mmHg and 12 mmHg, respectively. On the physical examination, the patient exhibited vesicles on her right forehead, scalp, and upper eyelid. Slit lamp biomicroscopy revealed conjunctival hyperemia and mild corneal erosion in the right eye. Inflammatory cells with Grade 1+ were present in the anterior chamber. On the fundus examination, multiple inflammatory lesions were observed at the posterior pole, along with exudative detachment of the macula (Fig. 1). The left eye showed no abnormalities. Choroidal folds with an irregular overlying retinal pigment epithelium (RPE) and hyper-reflective spots in the choroid layer were observed on optical coherence tomography (OCT). Fluorescein angiography (FA) presented a stippled pattern of hyperfluorescence in the late phase. A mixed pattern of hypo- and hyperpermeability was observed in both the early and late phases of indocyanine green (ICG) angiography. Polymerase chain reaction analysis of intraocular fluid showed positivity for VZV and negativity for herpes simplex type 1/2 and cytomegalovirus. Other laboratory examinations, including blood cell count and examinations for liver and renal function and angiotensin converting enzyme, showed normal findings. Serology for syphilis, toxoplasmosis, and human immunodeficiency virus (HIV) yielded negative findings. The patient was diagnosed with VZV-associated chorioretinitis and admitted. Intravenous acyclovir was immediately initiated. Oral prednisolone (30 mg) was added for relieving the inflammatory reaction and was slowly tapered. Topical ganciclovir ointment was applied for faster intraocular penetration and topical steroids were also used. After 2 weeks of treatment with intravenous acyclovir, the lesion resolved, with RPE undulation, and the visual acuity in the right eye improved to 20/20.

## Discussion

We described an unusual presentation of VZV-associated uveitis with exudative retinal detachment in an elderly woman. Chorioretinitis with exudative detachment of the macula resolved after 2 weeks of treatment with intravenous acyclovir and oral steroids. The manifestations of herpetic uveitis with posterior segment involvement may vary from ARN to milder non-necrotizing uveitis, [3] and there are a few reports on non-necrotizing variants [3–5]. Chorioretinitis is an unusual form of non-necrotizing herpetic uveitis, which was reported by Meenken et al. in two boys [5]. To our knowledge, no report has described VZV-associated chorioretinitis using serial OCT images obtained during the course of resolution.

OCT images of the chorioretinal lesion in the present case revealed RPE undulation with choroidal folds, hyper-reflective spots in the choroidal layer, and subretinal fluid. These findings resolved almost completely after 2 weeks of treatment with intravenous acyclovir. However, disruption of the inner segment–outer segment junction was observed after resolution. The final visual acuity in the right eye was 20/20 as the lesion was extrafoveal; however, permanent vision loss may occur if the foveal center is involved.

In the present case, FA showed hypofluorescence in the early phase and a stippled staining pattern in the late phase. These findings were similar to those in a previous report by Le et al. [6] ICG angiography, however, showed prominent hyperpermeability mixed with hypopermeability; these findings differ from those in the previous report. Fundus autofluorescence initially revealed decreased reflectivity due to blocking by subretinal fluid. After treatment, the area of decreased intensity was surrounded by increased margin intensity due to atrophy of RPE.

The mechanism underlying chorioretinitis in patients with VZV infection remains unclear. Bodaghi et al. hypothesized that non-necrotizing variants are retinal equivalents of stromal keratitis, whereas necrotizing herpetic retinopathies are equivalents of epithelial keratitis [4]. Host cellular immunity, rather than pathogen factors, may play a role in the development of viral uveitis. However, this hypothesis needs further investigation.

A differential diagnosis of progressive outer retinal necrosis (PORN) would be necessary in the present case. PORN occurs in severely immunocompromised patients and is characterized by multifocal deep retinal opacifications in the posterior pole that progress rapidly to confluence [1, 7]. This patient was immunocompetent with a normal blood cell count and was negative for HIV. The location of the lesion on OCT images was in the choroid and RPE layer, not in the outer retina. Moreover, the lesions remained discrete rather than confluent throughout the follow-up period. Although skin lesions were a major clue to suspect a viral origin in the present case, intraocular fluid analysis is important for differentiating longstanding uveitis, particularly in patients who are not responding to immunomodulatory treatments.

Intravitreal foscarnet injection in conjunction with systemic antiviral therapy for the management of ARN has been reported to be beneficial for decreasing the risk of severe vision loss and the incidence of retinal detachment [8]. Although the clinical course of VZV-associated chorioretinitis is milder than that of ARN, the adjunctive use of intravitreal foscarnet may also play a beneficial role in its treatment.

## Conclusions

In conclusion, the findings from this case suggest that VZV-associated posterior segment infection may present as multifocal chorioretinitis. Intraocular fluid analysis is crucial for the early detection of atypical posterior uveitis with an infectious origin.

## Abbreviations

ARN: Acute retinal necrosis
FA: Fluorescein angiography
ICG: Indocyanine green
OCT: Optical coherence tomography
PORN: Progressive outer retinal necrosis
RPE: Retinal pigment epithelium
VZV: Varicella zoster virus

## References

[1] Engstrom RE, Holland GN, Margolis TP, Muccioli C, Lindley JI, Belfort R, Holland SP, Johnston WH, Wolitz RA, Kreiger AE (1994). The progressive outer retinal necrosis syndrome. A variant of necrotizing herpetic retinopathy in patients with AIDS. Ophthalmology.

[2] Margolis R, Brasil OF, Lowder CY, Smith SD, Moshfeghi DM, Sears JE, Kaiser PK (2007). Multifocal posterior necrotizing retinitis. Am J Ophthalmol.

[3] Wensing B, de Groot-Mijnes JF, Rothova A (2011). Necrotizing and nonnecrotizing variants of herpetic uveitis with posterior segment involvement. Arch Ophthalmol.

[4] Bodaghi B, Rozenberg F, Cassoux N, Fardeau C, LeHoang P (2003). Nonnecrotizing herpetic retinopathies masquerading as severe posterior uveitis. Ophthalmology.

[5] Meenken C, Rothova A (2013). Varicella-zoster virus-associated multifocal chorioretinitis in 2 boys. JAMA Ophthalmol.

[6] Le TD, Weisbrod D, Mandelcorn ED (2015). Chorioretinitis with exudative retinal detachment secondary to varicella zoster virus. Canadian journal of ophthalmology.

[7] Forster DJ, Dugel PU, Frangieh GT, Liggett PE, Rao NA (1990). Rapidly progressive outer retinal necrosis in the acquired immunodeficiency syndrome. Am J Ophthalmol.

[8] Schoenberger SD, Kim SJ, Thorne JE, Mruthyunjaya P, Yeh S, Bakri SJ, Ehlers JP (2017). Diagnosis and treatment of acute retinal necrosis. Ophthalmology.

